# Extraordinarily Rare Isolated Intraperitoneal Urinary Bladder Rupture in Blunt Abdominal Injury Without Pelvic Fracture: An Aide-Mémoire

**DOI:** 10.7759/cureus.28576

**Published:** 2022-08-30

**Authors:** Dzemail Detanac, Nebojsa Filipovic, Ilker Sengul, Eldin Zecovic, Safet Muratovic, Demet Sengul

**Affiliations:** 1 General Surgery, General Hospital Novi Pazar, Novi Pazar, SRB; 2 Urology, General Hospital Novi Pazar, Novi Pazar, SRB; 3 Endocrine Surgery, General Surgery, Giresun University Faculty of Medicine, Giresun, TUR; 4 Gastroenterology, General Hospital Novi Pazar, Novi Pazar, SRB; 5 Pathology, Giresun University Faculty of Medicine, Giresun, TUR

**Keywords:** histopathology, surgical pathology, pathology, bladder rupture, rupture, urinary bladder, bladder, emergency, general surgery, surgery

## Abstract

Isolated urinary bladder rupture caused by blunt abdominal trauma is an infrequent injury, with less than 2% of all cases. It is most often found in traffic accidents and is usually associated with pelvic fractures. While an extraperitoneal bladder injury is mostly treated conservatively, an intraperitoneal one is mostly treated surgically. We present a 54-year-old obese man injured in a traffic accident, with an intraperitoneal rupture of the bladder after blunt abdominal trauma with no signs of pelvic fracture and without signs of traumatic injury to the abdominal organs. The vignette case constitutes a bladder injury that was treated surgically and sutured in two layers with absorbable thread, with the placement of a urinary catheter. The patient recovered without complications and was discharged from the ward on the seventh postoperative day. Of note, the clinical presentation of a bladder rupture can be manifested as a nonspecific lower abdominal pain or with symptoms of an acute abdomen and that is why imaging plays a major role in the diagnosis and further treatment procedures. Surgical treatment of such injuries can be performed laparoscopically or as open surgery. To this end, rapid diagnosis of bladder rupture is necessary because it can lead to complications that endanger patients if overlooked.

## Introduction

Blunt abdominal trauma is one of the leading causes of mortality and morbidity of all abdominal injuries. Bladder contusion is known as the most common type of bladder injury in abdominopelvic blunt trauma. However, isolated traumatic bladder rupture is infrequent and accounts for up to 2% of all cases [1]. They most often occur in motor vehicle accidents (MVAs) and are mostly associated with pelvic fractures. Full bladders are more susceptible to injury, and the dome of the bladder is the weakest part of the organ, which is the most susceptible anatomic location to injury [2]. Bladder rupture (BR) can be intraperitoneal, extraperitoneal (mostly associated with pelvic fracture), and combined intra and extraperitoneal injury. Approximately, 10% of cases are not associated with pelvic fractures, among those, less than 2% with isolated BR in blunt abdominal trauma [1]. While intraperitoneal injuries have been reported as mostly treated surgically successfully, most extraperitoneal ruptures can be cured via non-surgical modalities [3]. It is critical for general surgery clinicians to stay vigilant for the growing spectrum and clinical presentation of BR to ensure appropriate clinical care and management for the entity. Our vignette case possessed an isolated intraperitoneal bladder rupture caused by a blunt abdominal injury in an MVA, without a pelvic fracture.

## Case presentation

**Figure 1:**
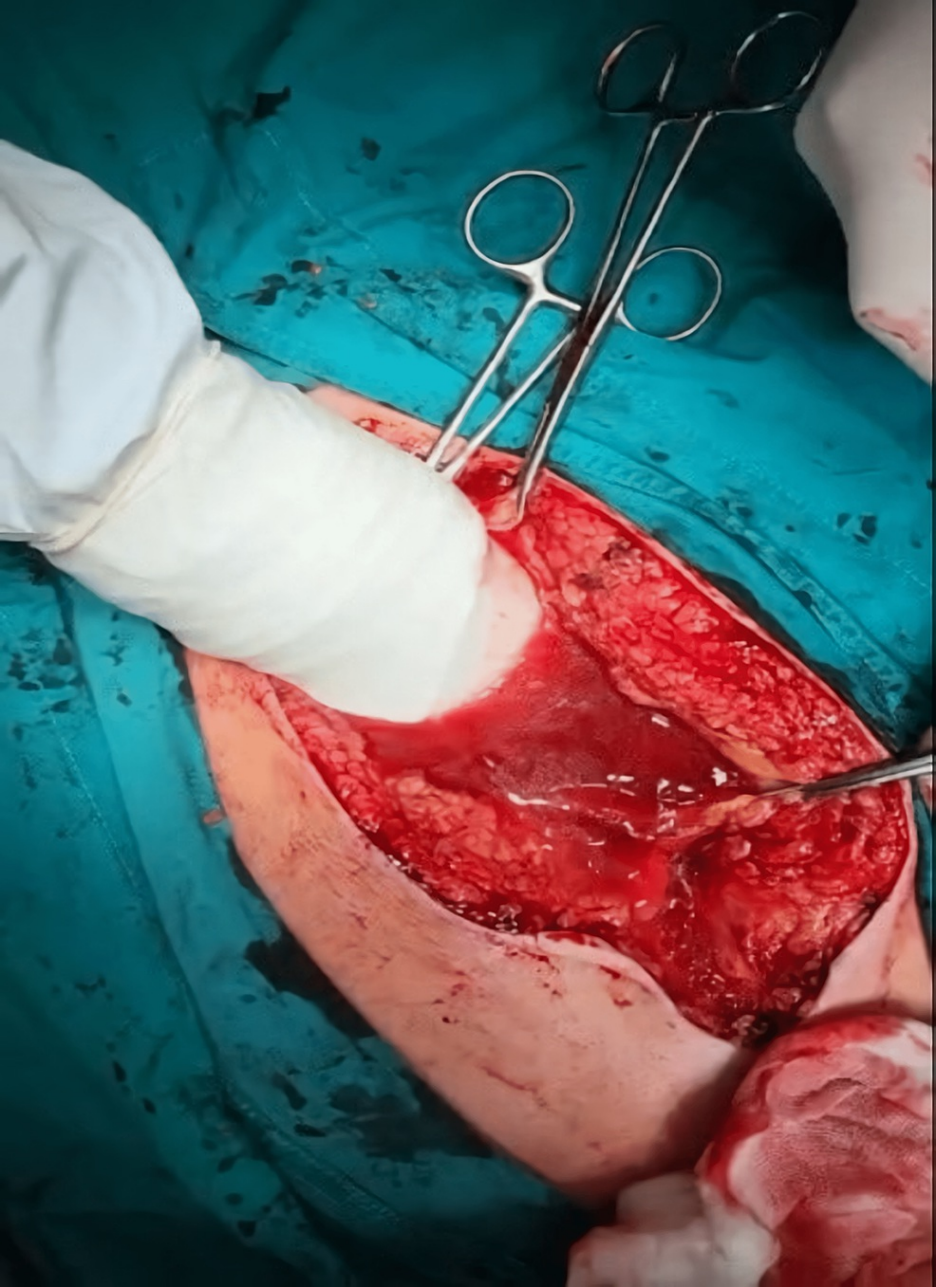
Intraoperative photograph: intraabdominal free fluid

**Figure 2:**
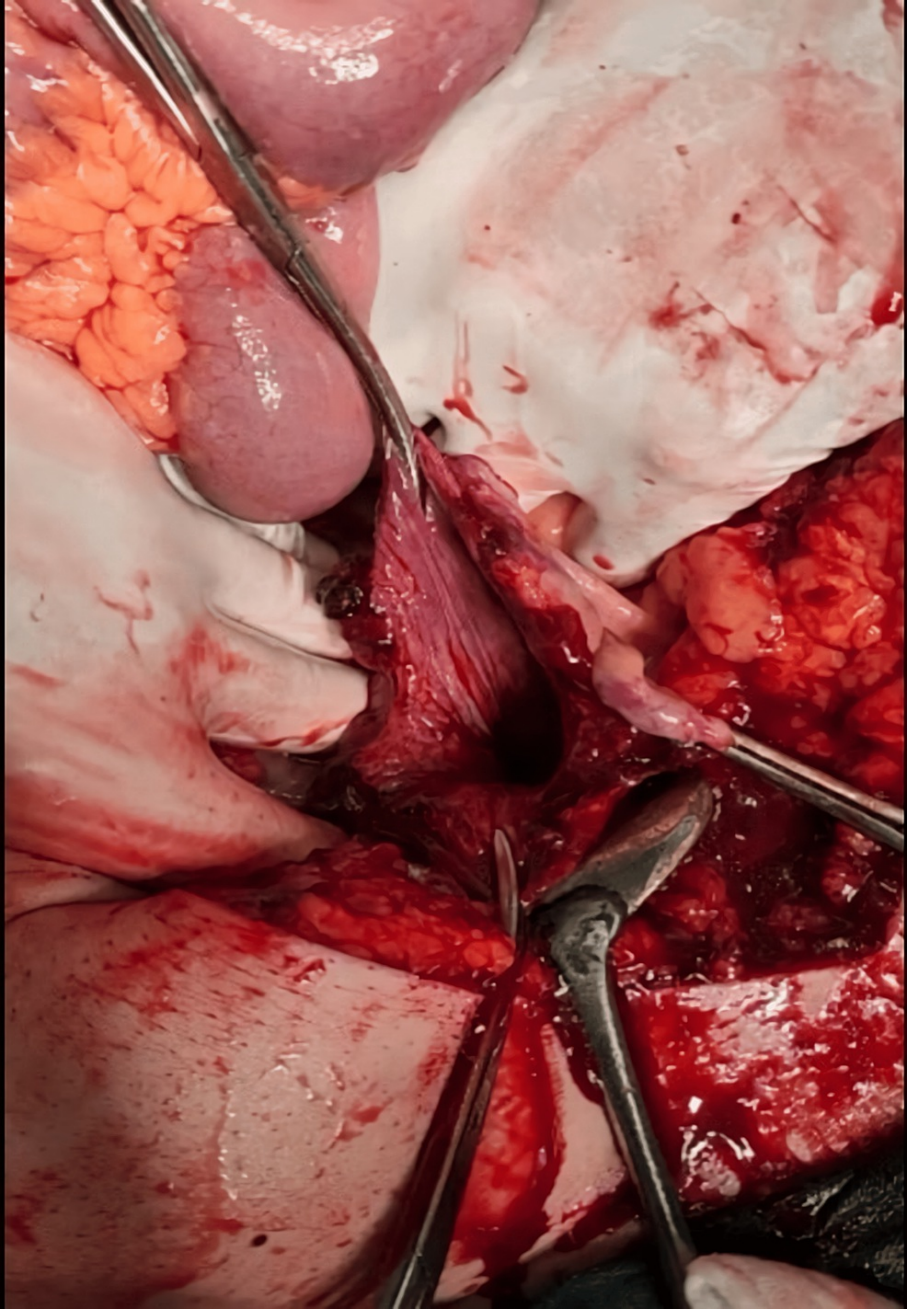
Intraoperative photograph: the ruptured bladder

**Figure 3:**
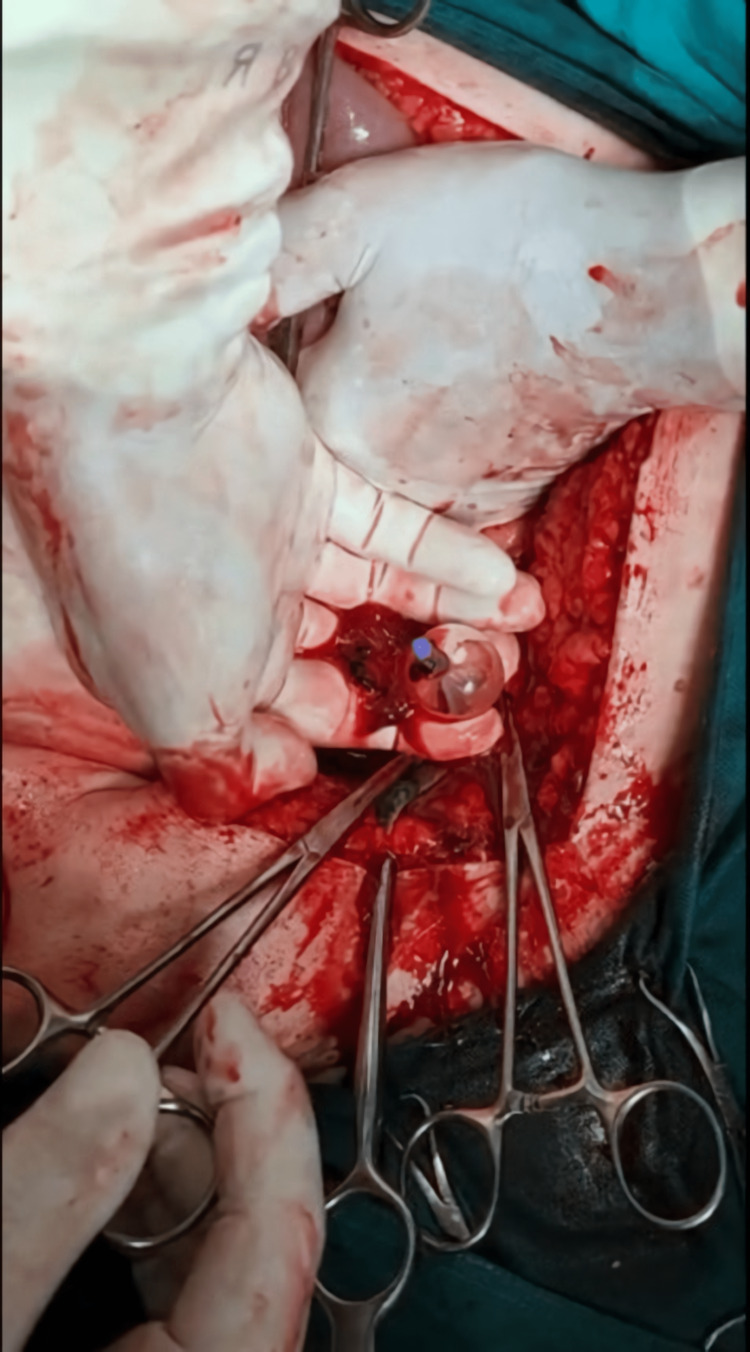
Intraoperative photograph: the ruptured bladder with a catheter inside it

A 54-year-old obese man, the driver of a car who was wearing a seat belt, was admitted to the Department of General Surgery due to an injury sustained in a traffic accident when he hit the car in front of him at a high speed in his journey. On admission, he was conscious, and communicative, without any information about head injury, with abdominal pain present. Inspection revealed an infraumbilical small subcutaneous hematoma of the anterior abdominal wall, 2x3 cm in size approximately. His medical history was positive for diabetes mellitus. He was in a hemodynamically stable, normotensive, and tachycardic status. On the chest X-ray, no signs of traumatic injuries were recognized. The abdominal ultrasound exhibited the presence of a small amount of free fluid adjacent to his bladder areas, without signs of a pelvic fracture on the plain radiographs. The laboratory analyses indicated the white blood cell count, 17.6x109/L; red blood cell count, 3.9x1012/L; hemoglobin 110 g/L; serum creatinine 164 µmol/L; and serum glucose 11.2 mmol/L. A urinary catheter was placed and a small amount of urine with blood was obtained. One hour after admission to the Department of General Surgery, his general condition worsened, the patient developed severe diffuse abdominal pain, and his abdomen was distended with diffuse tenderness. A non-contrast computed tomography (CT) of the abdomen and pelvis revealed the simple peritoneal fluid within the upper abdomen and pelvis with a suspected rupture of the urinary bladder without signs of traumatic damage to other organs in the abdominal cavity. Afterward, an emergent exploratory laparotomy was performed and upon opening the abdomen, a large amount of free fluid, mainly clear and slightly bloody, was recognized (Figure 1). In addition, a rupture of the peritoneal dome of the urinary bladder was identified intraoperatively (Figures 2, 3) without damage to other intra-abdominal organs. The urinary bladder was surgically sutured in two layers. He had an uneventful postoperative period with the laboratory parameters within reference values and was discharged from the ward on the seventh postoperative day. No complication has been recognized during his outpatient follow-up in the next three weeks with complete recovery.

## Discussion

Traumatism is a current topic in modern society and adequate treatment of traumatized patients is still a crucial challenge today [4]. Blunt abdominal injuries, in the 3rd place, right after head and chest traumas can cause injury to both the solid organs, mostly the liver and spleen, and the intestines. Because of its anatomical location in the pelvis, the bladder is well protected against external forces, injuries which represent only up to 2% of all blunt abdominal trauma cases [5].

Mechanisms of the bladder injury may occur in different ways such as i) direct blunt trauma to a distended bladder, ii) high-energy blunt abdominal trauma, which can lead to pelvic fracture and bladder injuries, iii) penetrating and iatrogenic injuries, frequently with a motor vehicle accident (MVA) or motorcycle collisions (45%), falls from altitudes (20%), and auto-pedestrian collisions [2]. Up to 90% of bladder injuries in blunt abdominal trauma are often associated with concomitant pelvic fractures. Isolated traumatic BR is rare, but extraperitoneal rupture is frequent, accounting for 80% of the cases [3,6]. Of note, early recognition of bladder injury is crucial for proper intervention. The present case was a seatbelt-restrained driver with an intraperitoneal BR without pelvic fracture. As such, it is still sometimes difficult to assess the clinical presentation of a blunt abdominal injury, which is why imaging plays a major role in the diagnosis and further treatment procedures. Last but not least, BR clinical presentation can be manifested as a non-specific lower abdominal pain or with symptoms of an acute abdomen.

Even ultrasonography (US) is a very widespread diagnostic method, has a limited role, and is a preliminary investigation in abdominopelvic blunt trauma. Herein, a free fluid on the abdominal US after abdominal trauma represents a positive finding but is unreliable in distinguishing types of free fluid like urine, blood, etc. The method of choice in the diagnosis of blunt abdominal trauma is CT, which allows the detection of both parenchymal and hollow organ injury, in hemodynamically stable cases [5]. Non-contrast CT scans can identify simple fluid ascites but often cannot identify a laceration on the bladder itself. Due to anamnesis obtained data on a suspected allergy to a contrast during a previous CT imaging (the patient previously underwent a CT diagnostic procedure due to a suspicious lesion in his lungs), a non-contrast CT scan was performed. Plain film cystography and CT cystography are specific and highly sensitive for BR. However, these diagnostic modalities were not appropriate in our case because those procedures require additional time and because of developing the signs of acute abdomen and diagnosed free intraperitoneal fluid patient required emergency exploratory laparotomy. Anecdotally, delayed diagnosis in emergency conditions leads to more-inflammatory alterations and irreversible histopathologic changes in the relevant tissues and organs [7,8]. Last but not least, emergency surgery remains significant in the aforementioned era, globally [9-13].

## Conclusions

Intraperitoneal BR requires surgical exploration, mostly through laparotomy, because they are usually more serious injuries that possess a higher risk for infection with a higher associated risk of morbidity and mortality compared to extraperitoneal BR. The site of the rupture is usually treated by suturing the bladder in one or two layers with absorbable surgical sutures. Laparoscopic repair of an isolated intraperitoneal BR can be an alternative to laparotomy in hemodynamically stable trauma cases with no other intraabdominal injury. Isolated extraperitoneal BR can be managed nonsurgically with urinary catheter drainage for up to three weeks. To date, emergency surgery remains significant in this era, globally. Awareness of this entity would allow an earlier approach and at­tempt to mitigate the relevant high morbidity and mortality rates, which will be vital for providers in the management of this issue.
